# Automated major psoas muscle volumetry in computed tomography using machine learning algorithms

**DOI:** 10.1007/s11548-021-02539-2

**Published:** 2021-12-20

**Authors:** Felix Duong, Michael Gadermayr, Dorit Merhof, Christiane Kuhl, Philipp Bruners, Sven H. Loosen, Christoph Roderburg, Daniel Truhn, Maximilian F. Schulze-Hagen

**Affiliations:** 1 Institute of Imaging and Computer Vision, RWTH Aachen, Aachen, Germany; 2 Department of Diagnostic and Interventional Radiology, University Hospital RWTH Aachen, Aachen, Germany; 3 Medical Faculty of Heinrich Heine University Düsseldorf, Clinic for Gastroenterology, Hepatology and Infectious Diseases, University Hospital Düsseldorf, Düsseldorf, Germany

**Keywords:** Opportunistic imaging, Psoas major muscle, Machine learning, Generative adversarial network

## Abstract

**Purpose:**

The psoas major muscle (PMM) volume serves as an opportunistic imaging marker in cross-sectional imaging datasets for various clinical applications. Since manual segmentation is time consuming, two different automated segmentation methods, a generative adversarial network architecture (GAN) and a multi-atlas segmentation (MAS), as well as a combined approach of both, were investigated in terms of accuracy of automated volumetrics in given CT datasets.

**Materials and methods:**

The bilateral PMM was manually segmented by a radiologist in 34 abdominal CT scans, resulting in 68 single 3D muscle segmentations as training data. Three different methods were tested for their ability to generate automated image segmentations: a GAN- and MAS-based approach and a combined approach of both methods (COM). Bilateral PMM volume (PMMV) was calculated in cm^3^ by each algorithm for every CT. Results were compared to the corresponding ground truth using the Dice similarity coefficient (DSC), Spearman’s correlation coefficient and Wilcoxon signed-rank test.

**Results:**

Mean PMMV was 239 ± 7.0 cm^3^ and 308 ± 9.6 cm^3^, 306 ± 9.5 cm^3^ and 243 ± 7.3 cm^3^ for the CNN, MAS and COM, respectively. Compared to the ground truth the CNN and MAS overestimated the PMMV significantly (+ 28.9% and + 28.0%, *p* < 0.001), while results of the COM were quite accurate (+ 0.7%, *p* = 0.33). Spearman’s correlation coefficients were 0.38, 0.62 and 0.73, and the DSCs were 0.75 [95%CI: 0.56–0.88], 0.73 [95%CI: 0.54–0.85] and 0.82 [95%CI: 0.65–0.90] for the CNN, MAS and COM, respectively.

**Conclusion:**

The combined approach was able to efficiently exploit the advantages of both methods (GAN and MAS), resulting in a significantly higher accuracy in PMMV predictions compared to the isolated implementations of both methods. Even with the relatively small set of training data, the segmentation accuracy of this hybrid approach was relatively close to that of the radiologist.

**Supplementary Information:**

The online version contains supplementary material available at 10.1007/s11548-021-02539-2.

## Introduction

Radiological examinations are performed for specific clinical purposes; however, especially cross-sectional imaging depicts a variety of body regions, that are not of primary diagnostic interest. Machine learning algorithms facilitate the development of “opportunistic imaging,” which is the process of utilizing unexploited, but potentially meaningful information in diagnostic imaging [1]. The volume of the major psoas muscle (PMM) has been identified to reflect the overall body muscle mass to a certain degree and is fully recognizable on abdominal CT scans [2, 3]; it is therefore an ideal muscle for three-dimensional segmentations. This becomes of interest in the assessment of sarcopenia, the age-related, progressive and generalized reduction of skeletal muscle mass, which is a condition that has been linked as a major risk factor for morbidity or mortality in multiple clinical conditions and complex surgical procedures [4–8].

As the process of manual muscle segmentation is highly time consuming and repetitive, it is destined to be automated or at least semiautomated. In the past, several attempts have been made to automate muscle segmentation based on CT images with varying degrees of success [9–12]. In recent research, convolutional neural networks (CNNs) have gained popularity and exhibit state-of-the-art performances in classification, segmentation and detection of objects in image processing [13]. However, state-of-the-art CNNs require a large amount of training samples to achieve reliable performance [9]. Among these, generative adversarial networks (GANs) have attracted the interest of the scientific community due to their architecture of two competing neural networks, a generator and discriminator, which can achieve reliable outputs in medical imaging [14]. In contrast to CNNs and GANs, atlas-based approaches achieve better results when only few annotated images are available. In the best-case scenario, even a single labeled training image can be sufficient to perform accurate automated segmentation. A major strength of these methods is the precise global alignment, which means that objects-of-interest are generally robustly identified. Furthermore, this approach can be easily applied to 3D image data without excessively increasing complexity. However, particularly in cases of high interpatient variability, this technique often leads to inaccurate segmentation of small details [10].

The aim of the study was to evaluate the performance of automated PMM-segmentations based on given CT datasets. As ground truth, manual segmentations of the bilateral PMM were used for training and testing of each of the above methods: the GAN and the MAS as well as a combined approach of both (COM).

## Materials and methods

### Image processing and segmentation

Ethical approval for this retrospective study was granted by the local institutional review board (EK 028-19). A total of 34 abdominal CT scans of patients with age between 20 and 80 years, acquired in the time between 01/2018 and 03/2018, were randomly selected from the hospital’s picture archiving and communication system (PACS). Inclusion criteria were venous contrast phase with a slice thickness of 5 mm. All CT examinations were performed in the Department for diagnostic and interventional Radiology, University Hospital RWTH Aachen, using either a 128-row multidetector CT scanner (Somatom Definition Flash; Siemens Medical Systems, Erlangen, Germany) or a 40-row multidetector CT scanner (Somatom Definition AS 40; Siemens Medical Systems, Erlangen, Germany). Tube voltage was 120 kV with a pitch of 0.6, and tube current was modulated according to Siemens CareDose4D. Images were reconstructed using filtered back projection. The anonymized CT-exams were extracted from the PACS, and the bilateral PMMs were manually segmented by a board-certified radiologist (6 years of experience) using ITK-SNAP (www.itksnap.org), an open-source software tool used to segment structures in 3D medical images [15]. Therefore, a total of 68 single psoas segmentations were available. Per sample, the number of slices varied between 76 and 139 with a scan matrix of 512 × 512 pixels.

### CNN-based segmentation

For CNN-based segmentation, a 2D generative adversarial network (GAN) architecture was applied to slices of the CT scans exhibiting a state-of-the-art approach for (biomedical) image segmentation [14]. This method consists of two adversarially trained networks, namely a generator, converting the input image into a segmentation mask, and a discriminator, assessing generated segmentation masks with respect to their fit to the underlying real images. The employment of a full 3D neural network approach that accesses an entire CT dataset was constrained by the complexity of the deep learning approach: The far greater amount of model parameters leads to non-convergence or overfitting with only limited training data available. To incorporate the intrinsic three dimensionality of the data, the 2D neural network was fed with information from the slice immediately above and below the active slice. In the following, this is referred to as 2.5D. Since the PMM in abdominal CT scans does not reach the most upper and lower sections of the exam, slices 2 to (*n*-1) were used for training purposes, as slices *1* and *n* share only one adjacent slice each. In the following, this approach is referred to as GAN. The loss formulation of the GAN approach can be summarized as an added sum of *L*_GAN_ and *L*_1_ where *L*_GAN_ forces the network to generate input–output combination that are realistic, while *L*_1_ forces similarity on pixel level. Expressed as formula, the losses can be formulated as:

$$ L_{{{\text{GAN}}}} \left( {G, \, D} \right) \, = E_{{\text{y}}} \left[ {\log \, D\left( {x,y} \right)} \right] + \, E_{{\text{x}}} [\log \left( {1 \, - \, D\left( {x,G\left( x \right)} \right)} \right] $$

$$\qquad L_{{{\text{L}}1}} \left( G \right) \, = \, E_{{{\text{x}},{\text{y}}}} \left[ {\left\| {y \, - \, G\left( x \right)} \right\|_{1} } \right] $$

with *E* being the expected value, *G* being the generator, *D* being the discriminator, x being the input image and y the output image. For the added sum, *L*_L1_ was weighted with a factor of 10.0 and *L*_GAN_ with a factor of 1.0.

### Training of the neural network

The training data were processed by concatenating each respective slice with the adjacent slice above and below (in *z*-direction). All 2D training and test samples were resized to 256 × 256 pixels. Input to the neural network was hence a matrix of size 256 × 256 × 3, which was subsequently fed into the GAN [11]. The number of epochs was set to 50. We used the U-Net [13] as generator and a PatchGAN as discriminator network [14]. The Adam optimization algorithm was applied based on the momentum term *ß* = 0.5, and the initial learning rate was set to 2 × 10^–4^.

### Multi-atlas segmentation

**Fig. 1:**
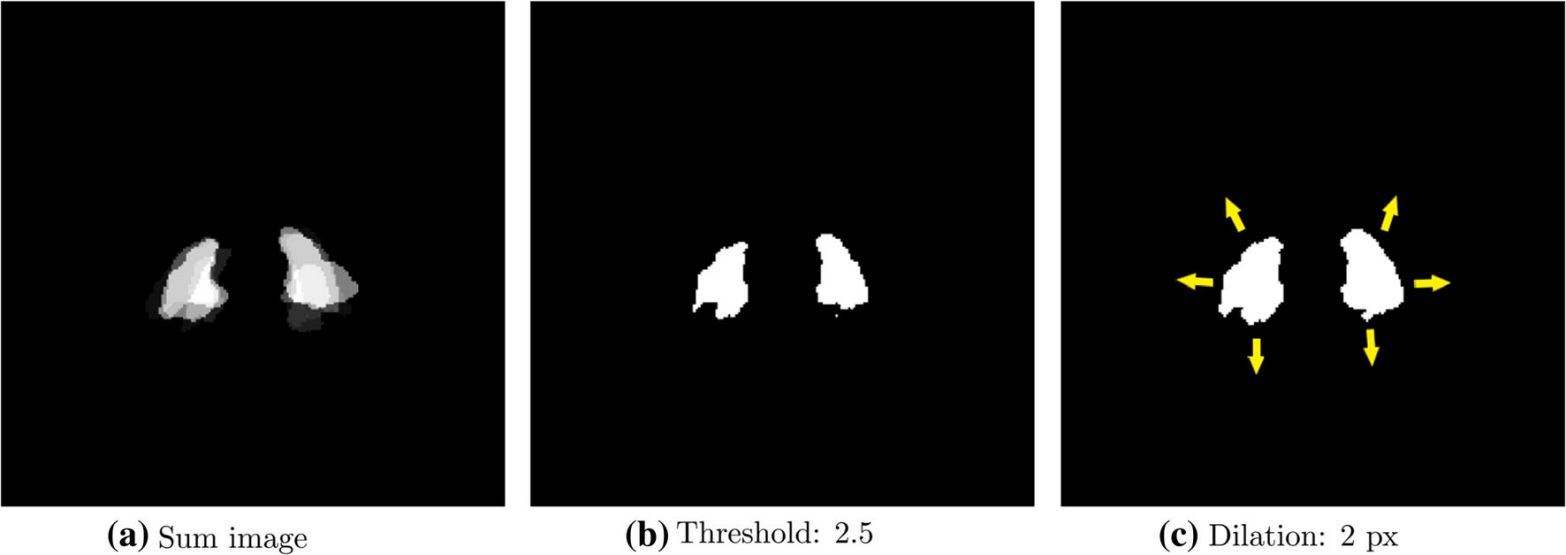
Process of generating the segmentation masks with the multi-atlas-based segmentation approach (MAS) and the additional dilation used for the combined approach (COM). First, the 5 best registered training samples were combined (**a**), and then, a threshold of n/2 was applied (**b**). Each registered annotation was interpreted as a binary image. By summing up all n binary images, voxel-values between zero and n were obtained. Setting the threshold to n/2, voxels were only classified as PMM if found in > 50% (majority voting) of the registered annotations. Subfigure (**c**) demonstrates the additional voxel dilation operation, which was exclusively executed in the MAS application in the COM

Atlas segmentation [16, 17] refers to a technique in which annotation masks are transferred from one image to another using image registration methods. Multi-atlas segmentation (MAS) [18] refers to a special kind of atlas segmentation approach which makes use of several reference images. The obtained annotation masks are finally merged to a single mask, potentially increasing robustness of the respective approach. For the purpose of this study, a dual-stage registration with annotated samples (atlases) was performed using Elastix-toolkit [12]. In the first stage, the global alignment was corrected by means of linear transforms, followed by correction of the local alignment by means of non-rigid transforms. This approach works directly on the full 3D volumetric data to encompass all global information. For this stage, intensity-based affine registration utilizing the mean squared error as loss metric was performed. For the sake of efficiency, this stage was performed on multiple resolution stages (original resolution and downscaled by factor eight, four, two and one). On the lowest scale, initial registration was performed. For the following scales, the output from the previous scale was used as initialization. Gradient descent was used for optimization. For the second stage, a B-Spline model was employed that utilized an adaptive stochastic gradient descent optimizer to maximize mutual information between target and source image. To achieve robustness, the segmentations achieved with several atlases were merged by means of priority voting. For that purpose, the five best atlas registrations were assessed by considering the minimized achieved energy (Fig. 1).

### Combined segmentation approach

To exploit the advantages of both, the GAN and the MAS approach, label maps for both approaches were computed separately and combined in a post-processing step. We made use of the fact that 3D atlas segmentation is generally globally accurate, but prone to inaccuracies with respect to small details. First, the proposed borders of the masks obtained by the MAS-based segmentation were extended (dilated) by 2 pixels to robustly cover the region of the psoas muscles, however, with the downside of being an overestimation of the PMMV. Secondly, these results were merged with the outputs generated by the GAN, creating the final masks. In detail, the two masks were combined by applying a voxel-wise *AND* operator, i.e., a voxel was classified as “psoas muscle” only if it matched in both methods, the extended (dilated) multi-atlas mask and the rather precise mask generated by the GAN. In the following, this approach is referred to as combined segmentation approach (COM).

### Experimental details

For evaluation purposes, a fivefold cross-validation was employed in which the dataset was stratified into an 80% training set and a 20% test set, such that images of patients used during testing had never been seen by the algorithm during training. By cycling through the data in this manner, test measures for all 34 patients were obtained. Segmentation performance of algorithms was measured using the Dice similarity coefficient (DSC). Mean and standard deviations of PMMVs were calculated for each scan and every algorithm and compared to the scores computed from the segmentations provided by the radiologist. PMMV was calculated by multiplying the number of voxels within the segmentation mask with the voxel volume. To determine whether DSC scores differ significantly, median and 95% confidence intervals, as well as the Spearman’s correlation coefficient and the (paired) Wilcoxon signed-rank test, were employed. The Wilcoxon test was chosen since the DSC scores of two approaches are corresponding but not Gaussian distributed. *P* values of < 0.05 were considered statistically significant.

## Results

**Fig. 2:**
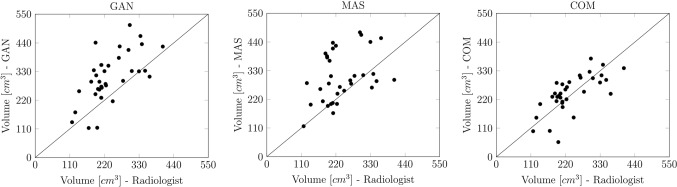
Scatter plots of the psoas major muscle volume (PMMV) as determined by the GAN (left), MAS (middle), and COM (right) vs. the radiologist. Left: Mean squared difference of the GAN-generated and manually segmented PMMV was 130.62 cm^3^, *p* < 0.001. Middle: Mean squared difference of the MAS-generated and manually segmented PMMV was 101.23 cm^3^, *p* < 0.001. Right: Mean squared difference of the COM-generated and manually segmented PMMV was 30.83 cm^3^, *p* = 0.33. *GAN: generative adversarial network architecture; MAS: multi-atlas-based segmentation; COM: combined approach of MAS and GAN*

34 CT examinations with a total number of 3887 slices were available for the study, ranging from 76 to 139 slices per CT. Mean PMMV over all patients was 239 ± 70 cm^3^ based on the radiologist’s segmentation, 308 ± 96 cm^3^ for the GAN (+ 28.9%, *p* < 0.001), 306 ± 95 cm^3^ for the MAS (+ 28.0% *p* < 0.001) and 243 ± 73 cm^3^ for the COM (+ 0.7%, *p* = 0.33). Mean squared differences of the generated versus the manually segmented PMMV and the corresponding Spearman’s correlation coefficient were 101.23 cm^3^ and 0.62 (*p* < 0.001) for the GAN and 130.62 cm^3^ and 0.38 for the MAS (*p* < 0.001), respectively. This indicates significantly differing deviations of the automated muscle segmentations from the ground truth. The mean squared difference of the PMMV and Spearman’s correlation coefficient of the COM versus the radiologist was 30.83 cm^32^ and 0.73, respectively, which demonstrated a robust alignment (*p* = 0.33). Scatter plots of automated versus the manual segmentations are demonstrated in Fig. 2.

**Fig. 3:**
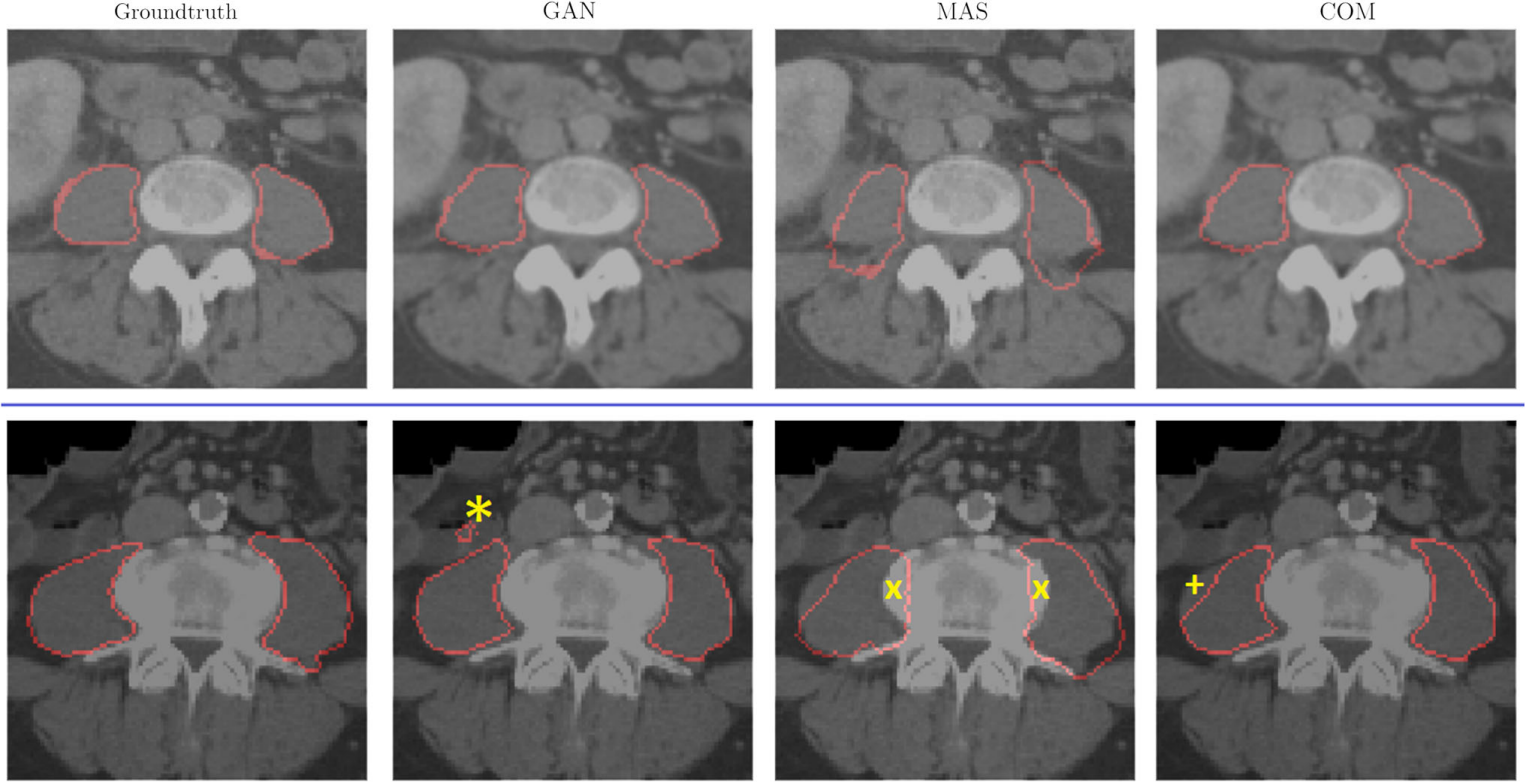
Exemplary segmentation results. The left column demonstrates the ground truth (manual segmentation of the radiologist) in comparison to results of the three automated approaches (GAN, MAS, COM). The first row demonstrates examples for each approach of accurate psoas major muscle (PMM) segmentations, and the bottom row demonstrates false positive and false negative misregistrations of the PMM for each approach. The GAN identified muscle boundaries quite effectively and was generally strong in differentiating the muscle from the surrounding retroperitoneal fat. A weakness was the tendency to identify false positive structures distant to the PMM (marked with *). In contrast, the MAS was relatively robust in the identification of the global alignment of the PMM; however, muscle boundaries were delineated quite inaccurately (marked with x). With COM, misregistrations of the MAS were partially transferred (marked with +). *GAN: generative adversarial network architecture; MAS: multi-atlas-based segmentation; COM: combined approach of MAS and GAN*

Median DSC for the GAN, MAS and COM was 0.73 [95%CI: 0.56–0.88], 0.75 [95%CI: 0.54–0.85] and 0.82 [95%CI: 0.65–0.9], respectively. Performance of the COM, as measured with DSC, was superior compared to both, the respective isolated approaches, GAN and MAS (each *p* < 0.001). A statically significant difference between the DSC of the combined approach with the radiologist’s segmentations was not detected (*p* = 0.3). Figure 3 shows exemplary segmentation results of the radiologist (ground truth) in comparison to GAN, MAS and COM, as well as examples of representative false positive and false negative segmentation results. Appendix 1 demonstrates a complete automated PMM segmentation by the COM.

## Discussion

We investigated the capability of three automated approaches to segment the PMMV in 34 randomly selected abdominal CT scans and compared the results to the manual segmentations of a board-certified radiologist. For that purpose, we applied a state-of-the-art 2.5D convolutional neural network (GAN), using a generative adversarial network structure (U-Net and PatchGAN), which was trained in a robust and efficient way to limit the amount of required manually annotated training data. We further evaluated a 3D multi-atlas-based segmentation method (MAS) as a conventional and rather robust image segmentation technique. Finally, the combined approach of both methods was also evaluated. The GAN identified the muscle boundaries quite effectively and was generally strong in differentiating the muscle from the surrounding retroperitoneal fat. While these fine details have a minor contribution to the overall muscle volume, they become of great importance for further analysis of muscle texture or fat content, such as the intramuscular fat fraction: even small misregistrations of the adjacent peritoneal fat might influence further applications significantly [19]. A weakness of the GAN was the tendency to identify false positive structures distant to the PMM (Fig. 3). These were easily distinguishable by humans as being separate objects. A full 3D approach could not be learned in the investigated setting due to availability of limited training data. We therefore employed a 2.5D approach by incorporating three consecutive slices as inputs to the GAN. In contrast, the MAS was relatively robust in the identification of the global alignment of the PMM, as compared to the GAN, which is a common strength of the approach [20]. However, muscle boundaries were delineated inaccurately, and muscle volume was generally overestimated, which is a relevant limitation.

Guidance of the 3D multi-atlas approach can potentially correct false positive detections of the GAN while maintaining the benefit of better accuracy in recognition of the muscle margins and border regions [21]. As expected, combining the accuracy of GAN-based methods in depicting small details and muscle margins and the robustness of the MAS was highly effective in this scenario. Taking into account that only 34 CT scans were used to train the algorithms, our results demonstrate the efficiency of the combined approach by exhibiting a relatively high accuracy of psoas-recognition, as expressed by the Dice coefficient of 0.82 [95%CI: 0.65–0.90]. In contrast to the isolated procedures (GAN, MAS), no significant difference was found regarding predictions of the COM versus manual segmentations of the radiologist. This was also reflected in the more accurate determination of PMMV. While the automatically determined PMMV for both isolated approaches (GAN and MAS) significantly overestimated the PMMV (+ 28.9% and + 28.0%, each *p* < 0.001), PMMV of the combined approach was comparable to the manually segmented volumes (+ 0.7%, *p* = 0.33). Thus, the automatic volume extraction of the presented combined approach resembled that of a human reader most closely.

It should be noted that there are of course other promising machine learning algorithms that allow psoas muscle segmentation, possibly with similar or even more accurate results, that were not investigated in this study. One example is SimCLR, a framework for contrastive learning [22]. Several other candidate approaches for deep learning-based segmentation have been proposed in the recent past [13, 23, 24]. Even though each method has advantages for certain settings, we decided to make use of the well-studied U-Net architecture [13] in an adversarial setting [14] to emphasize on learning shape priors. Regarding the combination of the two methods, another option would be given by an earlier fusion. The output of the MAS-based approach could also be used as input for the neural network. Since this would introduce several additional degrees of freedom, we chose the straightforward combination at the end of the pipeline. The results of the present study demonstrate the usefulness of a combination of two different approaches: a machine learning method and a traditional image segmentation method. In the end, this hybrid approach was able to achieve a high degree of precision compared to the manual segmentations of the radiologist.

There are some limitations of this study that need to be addressed. First, the relatively low number of CT scans made it difficult to robustly train a three-dimensional neural network, which is why a 2.5D approach was applied. However, the aim of this pilot study was to assess whether the proposed methods can be used in principle for the purpose of psoas muscle volumetry. Secondly, the retrospective selection of 34 randomly assigned CT scans from a single institution with an age distribution of 20–80 years might cause an underlying selection bias and thus could render the algorithms inapplicable to anatomical variants in certain patients. In addition, segmentations were not performed by multiple individuals, so inter-observer variability could not be determined. On the other hand, the fact that all segmentations were performed by the very same board-certified radiologist with 6 years of experience and that CTs were performed using uniform examination protocols may at least partially compensate for some of these limitations. Of course, a further validation in a dedicated test set is required for future studies.

## Conclusion

In conclusion, we demonstrate a promising dedicated approach to generate automated psoas major muscle volumetry with reasonable results, compared to manual segmentations of the radiologist. The fusion of a state-of-the-art deep learning approach with a robust conventional multi-atlas-based segmentation technique showed improved accuracy compared to both individual methods. Validation on a larger data set is currently ongoing. This approach could provide automated muscle volumetry for opportunistic imaging approaches in future studies, such as for the automated detection of sarcopenia.

## Supplementary Information

Below is the link to the electronic supplementary material. Complete automated psoas major muscle (PMM) segmentation by the combined approach of GAN and MAS in transverse plane in a CT-scan of the abdomen. GAN: generative adversarial network architecture; MAS: multi-atlas based segmentation;

Supplementary file1 (GIF 8501 kb)

## Data Availability

Due to data protection reasons, training and test data sets are only available to be used within the network of RWTH Aachen University Hospital in accordance with the requirements of the Ethics Committee.

## Declarations

### Code availability

The code of the software is currently not available as an open-source version.
